# Biological Width around One- and Two-Piece Implants Retrieved from Human Jaws

**DOI:** 10.1155/2014/850120

**Published:** 2014-06-23

**Authors:** Ricardo Judgar, Gabriela Giro, Elton Zenobio, Paulo G. Coelho, Magda Feres, Jose A. Rodrigues, Carlo Mangano, Giovanna Iezzi, Adriano Piattelli, Jamil Awad Shibli

**Affiliations:** ^1^Department of Periodontology and Oral Implantology, Dental Research Division, University of Guarulhos, Praca Tereza Cristina 229, 07023-070 Guarulhos, SP, Brazil; ^2^Department of Oral Implantology, Pucminas, Belo Horizonte, MG, Brazil; ^3^Department of Biomaterials and Biomimetics, New York University, 45 E 24th Street, New York, NY 10010, USA; ^4^Department of Stomatology and Biotechnologies, University of Chieti-Pescara, Via dei Vestini 31, 66100 Chieti, Italy

## Abstract

Several histologic studies regarding peri-implant soft tissues and biological width around dental implants have been done in animals. However, these findings in human peri-implant soft tissues are very scarce. Therefore, the aim of this case series was to compare the biological width around unloaded one- and two-piece implants retrieved from human jaws. Eight partially edentulous patients received 2 test implants in the posterior mandible: one-piece (solid implants that comprise implant and abutment in one piece) and two-piece (external hexagon with a healing abutment) implants. After 4 months of healing, the implants and surrounding tissue were removed for histologic analysis. The retrieved implants showed healthy peri-implant bone and exhibited early stages of maturation. Marginal bone loss, gaps, and fibrous tissue were not present around retrieved specimens. The biologic width dimension ranged between 2.55 ± 0.16 and 3.26 ± 0.15 to one- and two-piece implants, respectively (*P* < 0.05). This difference was influenced by the connective tissue attachment, while sulcus depth and epithelial junction presented the same dimension for both groups (*P* > 0.05). Within the limits of this study, it could be shown that two-piece implants resulted in the thickening of the connective tissue attachment, resulting in the increase of the biological width, when compared to one-piece implants.

## 1. Introduction

Biologic width (BW) is a physiologically formed and stable vertical dimension of the dentogingival junction that comprised the sulcus depth (SD), junctional epithelium (JE), and connective tissue attachment (CTA) [1]. In a similar manner to the teeth, dental implants have also a similar peri-implant soft tissue structure [2–4]. On the dental implants, the biologic width is influenced by several factors, and among them, the macrostructure, that is, one- or two-piece implants, seems to be most relevant [4]. The two-piece implants present a microgap placed at the crestal bone level, while one-piece implants have no gap at this region [5].

Early studies have shown [6–8] that the mucosal barrier around dental implants is composed by sulcular epithelium ranged between 1.5 and 2 mm and connective tissue between 1 and 2 mm. The oral epithelium presents an extension with a thin junctional epithelium facing the implant surface and extending about 1.64–2.35 mm from the mucosal margin. The dimension from the marginal portion of the peri-implant mucosa to the marginal level of bone-to-implant contact has been found to be about 3 mm.

Although the precise mechanism responsible for the crestal bone remodeling in 2-piece implants is currently under research, factors such as microbial colonization of the microgap, micromovements of the abutment, or an interruption of the blood supply when implants and abutments are placed transmucosally and have been also hypothesized [9–12]. Complementary to these factors, several studies have demonstrated a second discrete inflammatory cell infiltrate, additionally to the connective tissue lateral to the abutment fixture junction [5, 12, 13]. This infiltrated connective tissue (abutment ICT) was consistently separated from the plaque-associated infiltrate by a zone of normal, noninflamed tissue [5, 12–14].

As these data were obtained mainly from animal studies [5, 6, 8, 10, 12, 13] and only few studies have investigated human peri-implant soft tissues [2, 4, 14], the aim of this case series was to evaluate the biological width around one-and two-piece implants retrieved from human jaws.

## 2. Material and Methods

### 2.1. Patient Population

Eight partially edentulous subjects (5 women; 3 men) with a mean age of 56.40 ± 4.7 years, referred for oral rehabilitation of the posterior mandible with dental implants at the Oral Implantology Facility at the University of Guarulhos, were included in this study. Patients presenting mandibular bone height lower than 11 mm, smoking habit, pregnancy, nursing, or any systemic condition that could affect bone healing were excluded from this study. The Ethics Committee for Human Clinical Trials at University of Guarulhos approved the study protocol (CEP# 201/03) following the World Medical Association Declaration of Helsinki requirements. The protocol of the study was explained to each subject that signed the informed consent.

### 2.2. Experimental Implants

**Figure 1:**
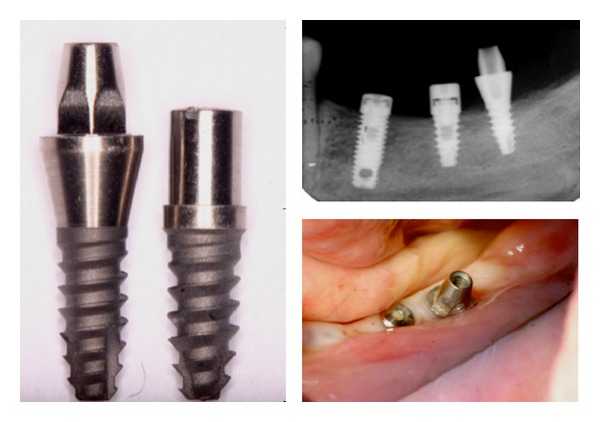
Clinical and radiographic view of the implants evaluated in the study.

Sixteen screw-shaped implants with sandblasted acid-etched surface, 3.3 mm diameter, and 8 mm length were used in this study. The implants were divided in 2 groups (*n* = 8): the one-piece implant group (solid implants that comprise implant and abutment in one piece) and the two-piece implant group (external hexagon with a healing abutment) (Figure 1).

All patients received an implant of each group (two implants were installed per patient). The implants were placed under the protocol previously reported [15–17]. Briefly, after crestal incision, mucoperiosteal flap was raised and conventional implant was placed in accordance with the surgical/prosthetic plan prepared for each patient. Afterwards, the experimental implants were placed in suitable areas, mostly in the second and third molar region, that is, posterior to the most distal conventional implant. The experimental implant recipient sites were prepared with a 2.8 mm diameter twist drill. All drilling and implant placement procedures were completed under profuse irrigation with sterile saline. If the experimental implant showed low primary stability, a backup surgical site was prepared. The experimental implants (one- and two-piece) were placed at the level of the alveolar crest. The flaps were sutured to allow nonsubmerged healing (Figure 1).

Amoxicillin was administered every 8 hours for 7 days, in order to avoid postsurgical infection. The sutures were removed 10 days post-operatively. Also, 0.12% chlorhexidine rinses were prescribed twice daily for 14 day in order to enable the postoperative dental biofilm control. Following the healing period of 4 months, the test implants and the surrounding tissues were retrieved with a trephine bur, and the specimens were fixed by immediate immersion in neutral formalin at 4%.

### 2.3. Specimen Processing and Histometric Analyses

The biopsies were processed to obtain thin ground sections as previously described (Precise 1 Automated System, Assing, Rome, Italy) [18]. The specimens were dehydrated in an ascending series of alcohol rinses and embedded in glycol methacrylate resin (Technovit 7200 VLC, Kulzer, Wehrheim, Germany). After polymerization, the specimens were sectioned lengthwise along the longer axis of the implant, using a high-precision diamond saw, to about 150 *μ*m, and ground down to approximately 30 *μ*m. Two slides were obtained from each implant and then averaged for each group. The slides were stained with basic fuchsin and toluidine blue.

The peri-implant tissue width was measured as follows:

- sulcus depth (SD): the distance between the mucosal margin (MM) and the most coronal point of junctional epithelium (CJE);
- junctional epithelium (JE): the distance between the most coronal point of JE and the most apical point of the JE;
- connective tissue attachment (CTA): distance between the most apical point of JE and the first bone-to-implant contact.

Therefore, the biological width (BW) was obtained after the sum of SD + MM + CTA.

Bone-to-implant contact (BIC%), defined as the amount of mineralized bone in direct contact with the implant surface, was also evaluated.

These measurements were performed using a light microscope connected to a high-resolution video camera and interfaced to a monitor and personal computer. This optical system was associated with a digitizing pad and a histometry software package with image-capture functionalities (Image-Pro Plus 4.5, Media Cybernetics Inc., Immagini & Computer Snc, Milan, Italy). A single trained examiner performed all histometric parameters.

The mean and standard deviation of histometric variables were calculated for each group. Nonparametric mixed models [19] were applied to evaluate the data clustered within the subject. The significance test was 2-tailed and conducted at a 0.05 level of significance.

## 3. Results

All 16 implants presented no mobility or clinical signs of infection after healing period. Bone density on the site of implant placement was almost D2 [20]. The retrieved implants showed healthy peri-implant bone. Osteocytes were present in their lacunae, although areas of woven bone could be distinguished. The newly formed peri-implant bone exhibited early stages of maturation. No gaps, marginal bone loss, or fibrous tissue were found surrounding retrieved implants.

**Figure 2:**
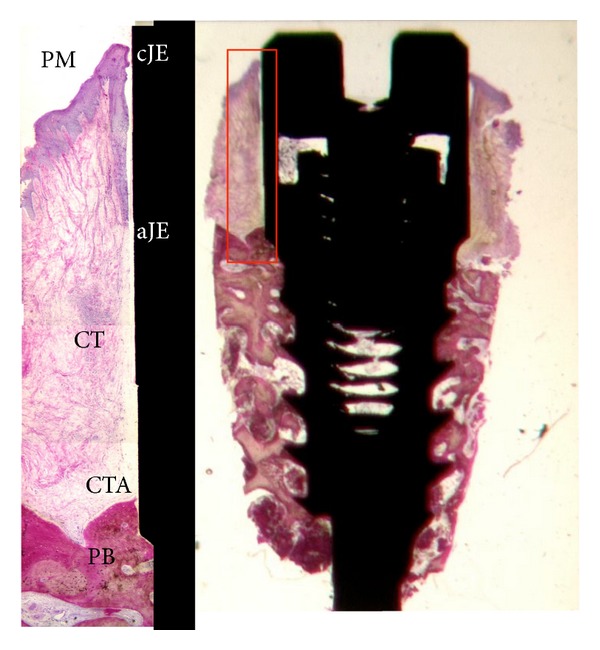
Photomicrograph of the ground section of the two-piece implant group (20x) and the high magnification of the biological width (100x).

**Figure 3:**
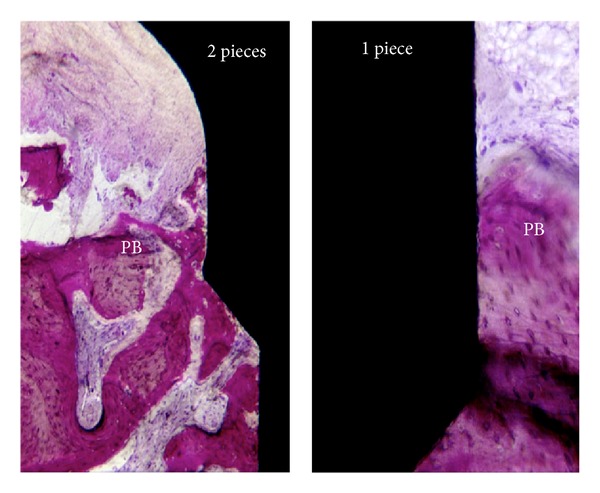
Photomicrographs of the one- and two-piece implant group (200x) near the first bone-to-implant contact. Note the disorganization around the peri-implant bone close to the microgap on the two-piece implant group.

The sulcular epithelium was composed of about 4–6 layers of parakeratinized epithelial cells. The junctional epithelium (JE) was composed of about 5–10 layers of epithelial cells. The middle and apical portion of the JE consisted of 3–5 layers of epithelial cells. No acute or chronic inflammatory cell infiltrate was present. Epithelial downgrowth was not depicted in any ground section (Figures 2 and 3).

In the abutment area of both groups, the connective tissue contained few blood vessels, and dense collagen fibers, oriented parallel to the longitudinal axis of the abutment, were present. Collagen fibers oriented in a perpendicular way and inserting directly contacting on the abutment surface were not observed in any of the specimens.

The mean dimensions of SD, JE, and CT for the 16 implants were reported in Table 1. An increase of the biological width's (BW's) dimension was observed, with mean values of 2.55 ± 0.16 and 3.26 ± 0.15 to the one- and two-piece implants group, respectively (*P* = 0.001). This difference was influenced by the CTA, since one-piece implants showed a CTA length average of 1.24 mm while two-piece implants presented 1.87 mm (*P* < 0.05). Sulcus depth (SD) and epithelial junction (EJ) presented no statistical significant difference between the groups. (*P* > 0.05). Moreover, BIC% showed no statistical difference between the groups as well.

## 4. Discussion

The current histological case series evaluated the influence of implant macrostructure (one- or two-piece implants) on human peri-implant soft tissues. Specifically, the biological width dimension was examined in implants retrieved from human jaws. The biologic width dimension ranged between 2.5 to 3.2 mm for one- and two-piece implants, respectively (*P* < 0.001). This difference was influenced by the connective tissue attachment, while sulcus depth and epithelial junction presented the same dimension for both groups (*P* > 0.05).

There is a lack of information regarding the histological features of supracrestal peri-implant soft tissue, since the present knowledge is basically constituted by animal studies data, using dogs and nonhuman primates [11] and some patient reports [3, 14, 21]. Although these data presents such important role in this field, sometimes the animal studies results cannot be faithfully transposed to the per-implant tissue behavior in humans [22]. A classical report using human teeth [1] showed that BW is a physiologically formed and stable dimension whose level is dependent upon the location of the alveolar bone crest. Around dental implants, BW determines the minimum dimensions to ensure adequate JE and CT to obtain an optimal seal and to provide protection from mechanical and external biological agents [23]. An external agent invading the BW would induce a response from the epithelium that migrates beyond this agent trying to isolate it [2, 3, 23]. The resulting bone resorption produces a reestablishment of the BW dimension. Regarding dental implants, the dimension of the BW was reported to be dependent on the presence/absence of a microgap and on the location of the microgap in relation to the bone crest [5–10].

Studies have reported the dimension of JE around implants, in animal studies, comprised between 1.16 mm and 1.90 mm [6, 8, 10, 11, 13], while JE around retrieved implants from human jaws ranged between 1.8 and 3.4 mm [4, 14], differing from the findings of this study that showed values similar to those reported in animal studies (~1.05 mm).

Connective attachment dimension, in animal models, ranged between 1.01 mm and 2.01 mm [10, 11, 13]. Loading conditions have also been reported to influence not only the dimension of JE but also the dimension of the CT. Previous canine model study [10] has shown that the CT dimension was significantly higher on unloaded implants when compared to different load conditions. The results of the present study could confirm a tendency for a larger size of the CT around unloaded implants. In fact, in human unloaded specimens, CT has been comprised between 1.8 mm and 3.4 mm [4, 14].

The supracrestal CT was, in animal studies, characterized by a 3D network of collagen fibers running in different directions [6, 8, 10]. In addition, several animal studies have reported a tight adaptation of the connective tissue to the abutment presenting a thin avascular and collagen fiber-rich, as a scar-like tissue characteristics [13, 23]. In the present specimens, the CT distant from the implant was composed by abundant collagen fibers, running in several directions and appearing to be functionally organized in a 3-dimensional network. Similar results have been reported in human studies [2–4, 14]. This differentiated network of fibers may have clinical relevance as a mechanical protection of the underlying bone [4]. These human histologic data are extremely valuable to validate and confirm those obtained from studies performed on animal models [8, 10, 11].

## 5. Conclusions

Therefore, within the limits of this histologic report, it could be suggested that the two-piece implant leads to a thicker biological width. These data must be carefully analyzed, and further prospective longitudinal studies are required to clarify the clinical relevance of these findings.

## Figures and Tables

**Table 1:** Mean ± standard deviation for the histometric variables of both groups. Wilcoxon rank test (*P < 0.05) (*n=8* subjects).

Histometric variables	1-piece	2-piece	*P* value
SD (mm)	0.33 ± 0.07	0.36 ± 0.12	0.98
JE (mm)	1.03 ± 0.06	1.05 ± 0.04	0.89
CTA (mm)	**1.24 ± 0.23**	**1.87 ± 0.20**	0.02*
BW (mm)	**2.55 ± 0.16**	**3.26 ± 0.15**	0.001*
BIC (%)	67.56 ± 4.56	66.45 ± 5.01	0.92

SD: sulcus depth; JE: junctional epithelium; CTA: connective tissue attachment; BW: biologic width; BIC: bone-to-implant contact.
